# Role of Epigenetic Mechanisms in Chronic Pain

**DOI:** 10.3390/cells11162613

**Published:** 2022-08-22

**Authors:** Daniela Mauceri

**Affiliations:** Department of Neurobiology, Interdisciplinary Centre for Neurosciences (IZN), Heidelberg University, 69120 Heidelberg, Germany; mauceri@nbio.uni-heidelberg.de

**Keywords:** epigenetic, chronic pain, DNA methylation, histone acetylation, histone methylation, non-coding RNA, gene expression

## Abstract

Pain is an unpleasant but essential-to-life sensation, usually resulting from tissue damage. When pain persists long after the injury has resolved, it becomes pathological. The precise molecular and cellular mechanisms causing the transition from acute to chronic pain are not fully understood. A key aspect of pain chronicity is that several plasticity events happen along the neural pathways involved in pain. These long-lasting adaptive changes are enabled by alteration in the expression of relevant genes. Among the different modulators of gene transcription in adaptive processes in the nervous system, epigenetic mechanisms play a pivotal role. In this review, I will first outline the main classes of epigenetic mediators and then discuss their implications in chronic pain.

## 1. Introduction

Acute pain has a relatively short duration, with different degrees of intensity. It is often caused by injury to tissues, and is essential for ensuring an organism’s survival by triggering a series of protective mechanisms. If pain persists over time, even after the healing process is complete or even in the absence of damage, it might take a deleterious turn and become chronic. Chronic pain does not have a protective function, but is instead harmful to the organism and is considered a pathology. Affecting a staggering percentage of the worldwide population, chronic pain is a severely debilitating condition with enormous socio-economic costs, reducing the quality of life and being accompanied by higher risk of mental health disorders [1,2]. Further, therapies to prevent or handle chronic pain are largely unsatisfactory [3]. Typical symptoms may be allodynia, defined as pain perception in response to innocuous stimuli, and hyperalgesia, in which noxious stimuli are perceived at greater intensity. Pain is, moreover, not only characterized by its sensorial component; it is a multidimensional experience additionally shaped by conscious aspects such as expectations, mood, memory and attention [4].

It is well established that chronic pain is not a simple extension in duration of acute pain, but rather involves distinct processes [5,6]. Several clinical studies, as well as research involving animal models of persistent pain, have shown that in the transition from acute to chronic pain some form of activity-dependent sensitization occurs in peripheral nociceptors, but also in neurons and glia cells in the central nervous system (CNS). Sensitization can happen in all anatomical compartments involved in pain processing. Sensitization at the level of nociceptors or dorsal root ganglia is generally named peripheral sensitization, while central sensitization is a term used to refer to higher excitability in CNS neurons, such as those of the dorsal horn spinal cord.

Sensitization is accompanied by structural and functional plasticity sustained via the modulation of gene transcription. Alterations in the expression of many genes have been reported both for nociceptors and CNS, and are thought to contribute to the transition from acute to chronic pain [7,8,9]. Synaptic activity-driven changes in the transcriptional profile of involved cells are key to shape plasticity in all forms of neuroadaptations. Gene expression might be under the direct control of transcription factors, but can also be under the influence of epigenetic modulators.

Epigenetic mechanisms indeed represent an important, additional, level of control of the regulation of gene expression. Epigenetic processes, comprising chromatin remodeling, DNA methylation and non-coding RNA, are receiving an increasing amount of attention within the pain research field. Not only has research on epigenetic mechanisms pushed forward our understanding on the molecular and cellular processes involved in maladaptation in pain chronicity, they have also sparked interest in the clinical community as potential new targets for the management of chronic pain. Already a decade ago, the scientific community had started addressing and discussing the then-emerging field of pain epigenetics [10]. The last years have seen the publication of an almost exponentially increasing number of articles on epigenetics and pain, contributing to the sharpening of what was initially a blurred hypothesis. However, a causally and mechanistically defined scenario explaining the role of epigenetic mediators in the development and maintenance of the different forms of chronic pain is still lacking.

This review will first outline the major epigenetic processes and their regulation to provide the reader with the necessary tools to become familiar with terminology and molecules, which are then discussed in the second part, highlighting key findings and open questions in epigenetic research in different forms of chronic pain.

## 2. Epigenetic Mechanisms

By definition, epigenetic mechanisms modulate gene expression without directly affecting the genetic code. The name results from the combination of “genetics” and “epi”—from the Greek word meaning “above”—to encompass the concept of changes not directly involving the DNA sequence but still modulating gene expression [11]. Historically, epigenetic marks had been defined as long-lasting or permanent, possibly self-regenerating and heritable across generations of cells or even organisms. The discovery that epigenetic processes take place also in adult neurons, which do not divide, brought into discussion this traditional view on epigenetics, and called for a broader interpretation. A few years ago, the term neuroepigenetics was therefore introduced to accommodate the concept that epigenetic mechanisms in the nervous system play very different roles from what they might do in imprinting, heritability or cell fate determination [12,13]. The most extensively studied epigenetic mechanisms are those regulating the state of chromatin and include DNA methylation and histone post-translational modifications (PTMs). The sum of all epigenetic marks makes up the epigenome [14] and plays a pivotal role in DNA condensation as it enables the packing of extremely long DNA molecules in a relatively tiny nucleus. Histone proteins are organized in octamers containing one H3-H4 tetramer and two pairs of histone H2A-H2B dimers [15]. A total of 146 bp of DNA are coiled around such structures and form the nucleosome. H1, a linker histone protein, mediates interaction between neighboring nucleosomes bringing to the formation of chromatin [16]. Chromatin can exist in two states: heterochromatin, closed and compacted, or euchromatin, more relaxed and therefore more compatible to transcription [17]. Epigenetic marks regulate the shift between the heterochromatin and euchromatin. Histones have a rather globular structure with a protruding N-terminal tail which is subjected to several PTMs such as acetylation, phosphorylation, methylation, ubiquitination, SUMOylation (Small Ubiquitin-like Modifier) and ADP ribosylation. There are a staggering number of enzymes regulating DNA methylation and histone PTMs, and they can be categorized according to their role. Writers are those enzymes adding a modification to DNA or histones, erasers remove the epigenetic chemical marks and, finally, readers identify and interpret the various modifications. In more recent years, non-coding RNAs have also started to be considered members of the epigenetic processes in lieu of their capacity to influence expression of genes without directly changing DNA genomic sequences.

### 2.1. DNA Methylation

The covalent addition of a methyl (CH3) group to DNA takes place on cytosines at the 5′ carbon position of the pyrimidine ring from the methyl donor S-adenosyl-methionine (SAM) [18]. The affected cytosines are predominantly located before a guanosine in so called CpG sites which tend to cluster into CpG islands, DNA regions of at least 200 bp positioned close to gene promoters.

**Figure 1:**
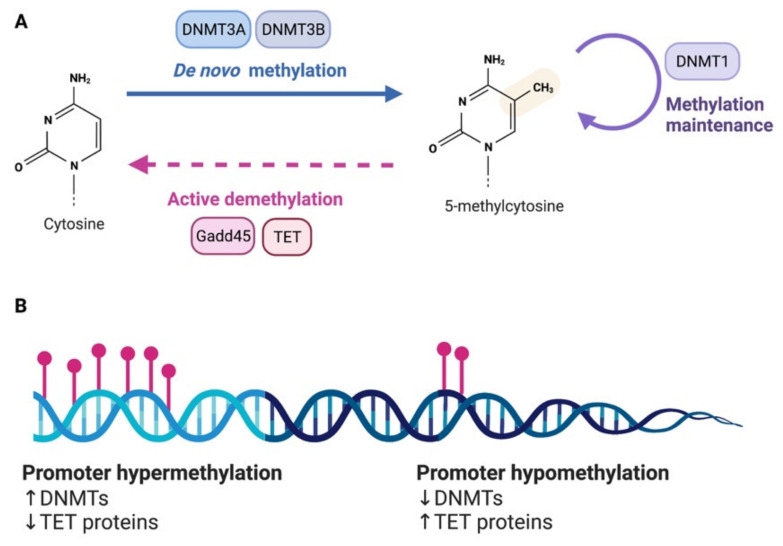
DNA methylation. (**A**) DNA methylation and demethylation are under the control of multiple enzymes. (**B**) Expression level of the responsible enzymes for DNA methylation can alter the methylation state of the promoters of specific genes. An increase in DNMTs will facilitate hypermethylation while a decrease of expression of DNMTs is associated to hypomethylation. On the contrary, a reduction of TET proteins, which mediate demethylation, will promote hypermethylation and an increase of TET can lead to hypomethylation.

DNA methylation is brought about by DNA methyltransferases (DNMTs). Several DNMTs exist in mammals and are categorized according to their preferred DNA substrate and methylation pattern. *De novo* DNMTs, DNMT3a and DNMT3b, add new methylation marks on previously unmethylated CpG sites. Maintenance DNMTs like DNMT1 instead are responsible for maintaining already established methylation marks [19] (Figure 1A). If one cytosine is methylated on a strand of DNA, this triggers DNMT1, which uses hemi methylated DNA as substrate, to methylate the corresponding cytosine on the opposite strand. Using this strategy, cells ensure self-perpetuation of the methylation mark in case of DNA damage or cell division [20]. DNMT1 has three prevalent isoforms (DNMT1s, DNMT1o, DNMT1p), and the *de novo* DNMTs also have multiple isoforms (i.e., DNMT3a1, DNMT3a2, DNMT3b1). In the case of DNMT3a, the variants originate from an intronic promoter located within the DNMT3a gene locus [21]. In addition to the three canonical DNMTs, there are two non-canonical family members—DNMT2, DNMT3L—which do not possess DNMT catalytic activity. DNMT2 acts predominantly as a tRNA methyltransferase [22] while DNMT3L increases the catalytic activity of DNMT3A and DNMT3B [23]. At the structural level, DNMTs generally feature an N-terminal regulatory domain followed by the catalytic domain positioned at the C-terminus. Further, they have domains for interaction with chromatin, DNA and other proteins involved in the regulation of gene transcription.

In light of the stability of the covalent bond underlying methylation, DNA methylation was previously considered to be permanent and immutable. However, DNA demethylation can take place in passive or active form. Passive DNA demethylation can be observed in the absence of maintenance DNA methylation during cell division. As neurons are postmitotic, DNA demethylation in these cells must be an active process and indeed several evidence have shown that DNA demethylation is a very dynamic process in the nervous system. Among epigenetic erasers implicated in DNA demethylation in neurons, we find the Growth Arrest and DNA Damage-inducible (GADD) 45 proteins (GADD45A and GADD45B) [24] and the ten–eleven translocation (Tet) family of dioxygenases [25] (Figure 1A,B). DNA demethylases appear to play a critical role in both adaptive and maladaptive processes in the nervous system [26,27].

Gene expression is modulated by methylated DNA via two main mechanisms; either the methyl-cytosines inhibit the binding of transcription factors due to steric hindrance or they are recognized by proteins featuring a methyl-binding domain (MBD), which, in turn, recruit proteins with chromatin remodeling capacities, transcription factors or repressors. The best studied and characterized reader of the DNA methylation epigenetic mark, methyl CpG binding protein-2 (MeCP2), has been implicated in the regulation of several neuronal functions both in development and adulthood [28]. Historically, DNA-methylation has been associated with compact chromatin and transcriptional inhibition. Indeed, methylation of cytosines can lead to the dissociation of transcription factors from their binding sites [29] and facilitate binding of methyl-CpG-binding proteins (MBPs), which can recruit co-repressors complexes [30,31]. This view has more recently been challenged by numerous studies indicating that depending on the genomic location, DNA methylation can also result in induction of gene expression. Indeed, it appears now clear that DNA methylation does not necessarily equal to repression of transcription [32]. One hypothesis, supported by a growing body of evidence, is that DNA methylation might put the genome in a permissive state to external stimulus-evoked responses [33,34,35].

### 2.2. Histone Modifications

**Figure 2:**
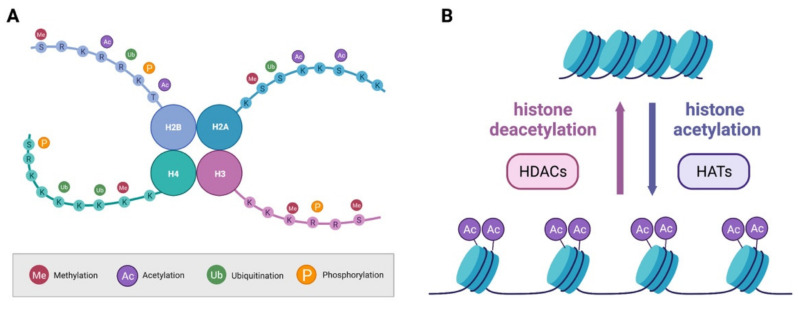
Histone PTMs and chromatin state. (**A**) Histone proteins can undergo several PTMs such as methylation, acetylation, ubiquitination and phosphorylation. (**B**) The balance between acetylation and deacetylation of histone proteins influences the structure of chromatin and is under the control of HATs and HDACs.

As outlined above, histone proteins undergo a plethora of PTMs on their protruding N-tails (Figure 2A). These modifications are reversible, and their status is controlled by enzymes with antagonistic functions who will add (writers) or remove (erasers) the modification. PTMs influences the interaction of histones with DNA and other proteins ultimately facilitating or opposing transcription. Methylation and acetylation are the most common and best studied PTMs of histone proteins and will be discussed in this section.

Lysine and arginine residues on histones can be methylated in multiple valence state. Arginine can accept one or two methyl groups while lysine residues can be mono-, di- or trimethylated. The different methylation state and pattern leads to activation or repression of gene transcription via alteration of chromatin structure by strengthening or weakening the DNA–histone interaction. The best characterized histone methylation mark is the one of histone 3 on lysine 4 (H3K4), particularly enriched at the transcription start site of actively transcribed genes. When trimethylated, this site prompts the loosening of chromatin and facilitates recruitment of transcription factors [36]. On the other hand, H3K27 is a hallmark of repression of gene expression. The family of methyltransferases comprises arginine and lysine methyltransferases, classified according to their substrate residue [37]. Like for DNA methylation, also histone methylation was long-considered a permanent epigenetic mark. However, it is now clear that histone demethylation is a very dynamic and fine-tuned process. There is a very large number of enzymes with demethylase activity, classified into two big families depending on their substrate and reaction mechanisms: lysine-specific histone demethylases (LSDs), of which LSD1 was the first demethylase to be ever identified, and Jumonji-C (JMJC) demethylases. For comprehensive molecular details of the complex and crowded histone demethylases and methylases scenarios, we remind to excellent reviews [38,39].

In addition to methylation, lysine residues on histone proteins can also undergo acetylation. The addition of an acetyl group to the positive charged lysine residue results in a decrease in the electrostatic affinity of histone proteins with the negatively charged DNA, shifting chromatin structure towards relaxation, and thus promoting transcription [40] (Figure 2B). Removal of the acetyl group is instead generally associated with a more compact state of the chromatin and inhibition of transcription. The acetyl group is transferred by histones acetyl transferases (HATs), such as p300/CBP (CREB (cAMP response element-binding) binding protein), using acetyl CoA as a cofactor (Figure 2B). It is known that HATs act not only on histones, but on a whole array of proteins, including non-nuclear ones, and are also thus named K-acetyltransferases (KAT) [37]. Like many other families of epigenetic regulators, KATs are numerous and divided into subgroups depending on their subcellular localization and structure [41]. Erasers of the acetylation mark are histone deacetylases (HDACs; Figure 2B).

**Figure 3:**
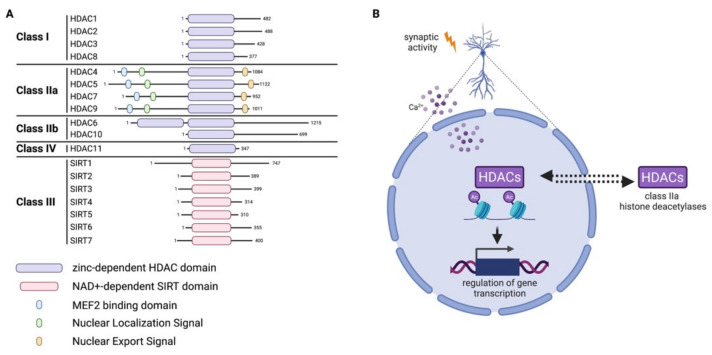
Histone deacetylases (HDACs). (**A**) Classification of histone deacetylases and sirtuins. The major domains of the different proteins are depicted. (**B**) Synaptic activity regulates the nuclear import or export of class IIa HDACs in neurons. This phenomenon ultimately modulates activity of these HDACs with important effects on gene transcription.

HDACs are a family of 18 proteins divided into two major groups, depending on their domains and use of cofactors: the classical zinc-dependent HDACs, comprising HDAC class I, II, IV; and the class III NAD-dependent sirtuins (SIRT 1-7) [42]. Class I comprises HDAC1, 2, 3, 8; class II is further divided into IIa (HDAC4, 5, 7, 9) and IIb (HDAC6, 10); and finally, the only member of class IV is HDAC11 (Figure 3A). Members of class IIa show a particular behavior which influences their activity, namely that they can shuttle between the nucleus and cytoplasm of cells in a signal-dependent manner [43,44]. In pyramidal neurons, the subcellular shuttling of class IIa HDACs is controlled by synaptic activity and nuclear calcium [45,46] (Figure 3B). In the CNS, the balance between the activity of HATs and HDACs, inducing or repressing gene transcription, controls developmental processes [47] and plays a role in aging and in neurodegenerative disorders [48,49] as well as in adaptive processes [50,51,52]. Readers of acetylated lysine on histone contain specific domains such as bromodomain (BRD), double plant homeodomain (PHD) fingers and Yeats domains [53].

### 2.3. Noncoding RNAs

The non-coding (nc) elements represent most of the human genome, as only about 1.5% of it encodes for proteins. These nc elements exert regulatory functions over the coding ones and have been implicated in numerous physiological functions. The ncRNAs are classified in two classes based on their length: short—less than 200bp—and long—more than 200bp—ncRNAs (lncRNAs). The short ones include microRNAs (miRNA), small interfering RNA (siRNA), short hairpin RNA (shRNA), small nuclear and nucleolar RNA (snRNA/snoRNA), transcription initiation RNA (tiRNA), ribosomal RNA (rRNAs) and piwi-interacting RNA (piRNA). LncRNAs include ncRNA expansion repeats, natural antisense transcripts and enhancer RNA intergenic ncRNA [54].

The best studied of the short ncRNAs class are the miRNAs, single-stranded, 19–22 bp long, which inhibit gene expression via triggering the degradation of specific mRNAs. miRNAs can target one or multiple mRNAs and have been shown to regulate up to 60% of all coding genes [55]. In the nervous system, their expression pattern depends on the brain region as well as the developmental stage. miRNAs have been implicated as key regulators in diverse adaptive processes such as structural and functional plasticity [56].

Similar to mRNA, lncRNAs are produced by RNA Polymerase II and then undergo processing. Particularly important for their activity appears to be their secondary structure, which defines the interaction with genomic DNA or proteins. Numerous lncRNAs have been identified, but only a limited amount of these have been experimentally defined in terms of their mechanism of action. Nevertheless, a scenario is emerging, picturing them as important regulators of gene expression with a variety of approaches: modification of chromatin, imprinting, RNA splicing, interaction with transcription factors, control of translation and trafficking from the nucleus [57].

## 3. Epigenetic Mechanisms in Chronic Pain

**Figure 4:**
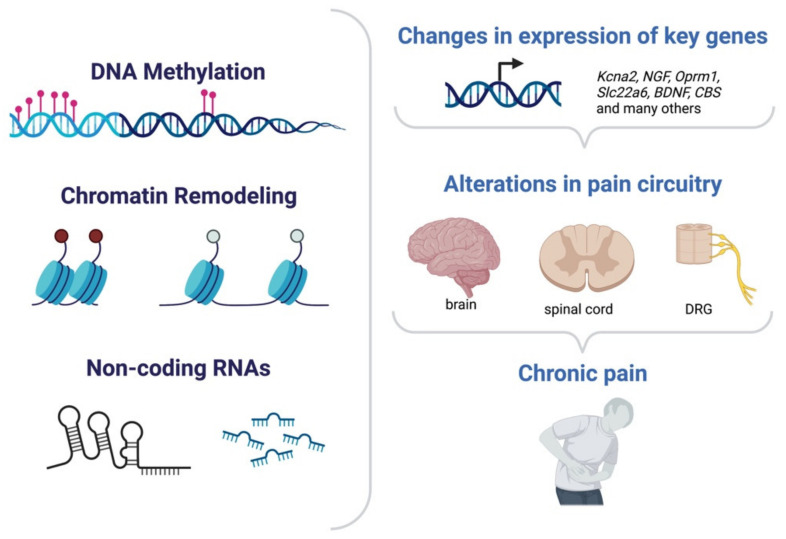
Epigenetic mechanism in chronic pain. Changes in DNA methylation level, chromatin structure or ncRNAs may affect gene expression of several different genes enabling long-lasting plasticity of the pain circuitry both centrally and peripherally and ultimately enabling chronic pain.

Induction or repression of gene expression sustains maladaptive changes in chronic pain. Such alterations in the transcriptional profile of cells have been found to be often under epigenetic modulation and have been detected across all the main anatomical regions involved in pain processing (Figure 4). The first site responsible for the detection of noxious stimuli, such as heat, cold, pressure, or acid, are peripheral sensory neurons. These bipolar neurons have their soma in dorsal root ganglia (DRGs) and their axon terminals located in the periphery and can transmit nociceptive inputs to the spinal cord, located in the CNS. Hyperexcitability of sensory neurons is one of the hallmarks of many forms of chronic pain. The second station in the pain processing route is the dorsal horn in the spinal cord which receives the input from the periphery and will ultimately relay the information to higher levels. The specific brain regions and circuits involved in the different pain modalities and in chronic pain remain to be defined. In the context of epigenetic modulation of transcription in pain chronicity, most studies indeed focused on either peripheral sensory neurons or neurons in the spinal cord, while only a fraction reported on epigenetic-mediated transcription in brain areas in chronic pain ultimately bringing to a better understanding of the sensory component of chronic pain in comparison to the emotional aspects. Mechanistically, epigenetic-regulated control of transcription supports chronic pain plasticity with different modes of action: it can (i) enable changes in the excitability of relevant circuits by modulating expression of key channels; (ii) induce or repress expression of postsynaptic receptors and signaling molecules; (iii) facilitate maladaptive structural plasticity for instance at the level of synaptic contacts or afferents; and (iv) alter the number of cells which will be recruited in the mediation of pain by affecting their responsiveness. The following paragraphs will discuss the main findings and mechanism of action of DNA methylation, histone methylation or acetylation and ncRNAs in chronic pain.

### 3.1. Chronic Pain and DNA Methylation

Changes in the global methylation state of DNA in spinal cord and DRGs in animal models of chronic neuropathic pain weeks after the injury have been observed and support the idea that methylation might be an important player in persistent pain [58,59]. In addition to alteration in the global methylation level of DNA, many studies detected increases in the methylation state in the promoter region of specific genes associated to chronic pain. Rodent models of neuropathic pain, in example, were associated to higher methylation of the promoters of the *Oprm1* (encoding mu 76 opioid receptor, MOR) and *Kcna2* (Potassium Voltage-Gated Channel Subfamily A Member 2) genes and, in agreement with the role of DNA methylation in repression of gene expression, their protein levels were decreased [60,61,62]. It is known that in neuropathic pain, opioid analgesic effects decrease, and an explanation might therefore be the epigenetic downregulation of MOR. Kcna2 expression level is considered a prominent aspect driving DRG neuronal excitability [63,64]. Variations in the methylation pattern of DNA in chronic pain are not strictly unidirectional towards an increase; hypomethylation of specific promoter regions was also observed. Such is the case for the promoter of the genes encoding for the chemokine receptor 3 (CXCR3) [65] or G Protein-Coupled Receptor 151 (GPR151) [66], which were both found to be less methylated in the spinal nerve ligation (SNL) mouse model of neuropathic pain. CXCR3 is a key player in the pathophysiological process of many inflammatory conditions and associated to pain modulation in DRG and spinal cord [67]. GPR151 has been associated to DRG hyperexcitability in neuropathic chronic pain [68]. Methylation changes are not specific to neuropathic pain, as different forms of chronic pain, like chronic inflammatory or visceral, were also accompanied by different methylation states [69,70,71]. Intra-plantar injection of rats or mice with complete Freund’s adjuvant (CFA) is one of the best characterized and used models of persistent inflammatory pain [72] and caused demethylation of the *NGF* (*Nerve Growth Factor*) [70] or *cystathionine-β-synthase* (*CBS*) [69] gene promoter regions. The detected changes in DNA methylation are implemented by the concomitant induction or repression of expression of DNMTs. Like what has been reported for the promoters of specific genes, changes in DNMTs expression profile happen in multiple pain states, in DRG as well as in the spinal cord, and can be bidirectional. DNMT3a is upregulated in both chronic constriction injury (CCI) and SNL rodent models of neuropathic pain [60,62,73,74,75,76]. These studies made no distinction between the DNMT3a isoforms, DNMT3a2 and DNMT3a1. The two isoforms have, however, quite different patterns of expression and activity in other areas of the nervous system. In the mouse hippocampus, expression of DNMT3a2 is under the control of synaptic activity and induced upon stimulation, while DNMT3a1 levels are unchanged [77]. In the context of chronic pain, it was shown that intraplantar delivery of CFA, to elicit persistent inflammatory pain, specifically induced DNMT3a2 in the dorsal spinal cord of mice leaving unaltered DNMT3a1, similar to what was described for the hippocampus [77]. In an opposite direction to that described for DNMT3a in most studies, namely chronic pain-associated induction, DNMT3b decreases under chronic pain conditions, and this reduction is considered responsible for the hypomethylation state of certain genes both in inflammatory and neuropathic pain [65,66,70]. The importance of DNA methylation in chronic pain is further supported by evidence pertaining the readers of this epigenetic mark. In the dorsal horn spinal cord, MeCP2 becomes phosphorylated, and thus inactivated, following inflammatory pain triggered by CFA treatment in rats [9]. Further, mice deficient in MBD1 showed alterations in hypersensitivity in neuropathic pain [78].

### 3.2. Chronic Pain and Histone Modifications

Methylation can also take place on histone proteins. In peripheral nerve injury, a model of neuropathic pain, the injury triggers the downregulation of different voltage-gated potassium channels. Ultimately, since their prime role is to inhibit the generation of action potential, their downregulation contributes to the typical hyperexcitability of the DRG neurons in chronic pain [63,64]. Histone di-methylation of the lysine 9 residue of histone 3 (H3K9me2) is greatly increased following nerve injury because of the upregulation of the histone methylase G9a. Two independent studies functionally linked the higher expression of G9a and H3K9me2 to the downregulation of voltage-gated potassium channels in rodents [79,80]. G9a belongs to a superfamily of histone methyltransferases [81] and seems to play an extensive role in pain modulation as it was also associated to changes in the expression of Oprm1 (encoding for the opioid receptor MOR) and cannabinoid receptors 1 and 2 (CB1 and CB2) following nerve injury in rats and mice [82,83]. As cannabinoid or opioid administration often loses efficacy over time in chronic pain patients, this finding represents a potential explanation for their pharmacological pattern. Both CB receptors have additionally been associated to modulation of inflammatory pain and, more generally, inflammation in diverse cell types [84,85,86,87]; it would therefore be interesting to assess the epigenetic regulation of expression of CB receptors under inflammatory pain conditions.

Beyond lysine methylation, arginine histone methylation has also been associated to pain; expression of the arginine-specific demethylase JMJD6 (Jumonji Domain Containing 6) is altered in a rat model of neuropathic pain [88].

Alterations in the level of histone acetylation were described for different models of pain including inflammatory and neuropathic [89,90]. Histone acetylation is under the opposing control of HATs and HDACs [40]. Several studies employed different HDAC inhibitors to promote analgesia in rodent models [91,92,93]. It was not possible, however, to describe a clear mechanistic scenario based on these studies as contradictory findings were reported [94]. In some cases, pharmacological HDAC inhibition ameliorated hypersensitivity while in others it worsened it [95,96,97]. Most likely, the discrepancies are to be ascribed to the fact that the used HDAC inhibitors are far from being specific and, in the best of cases, inhibit an entire subclass comprising of multiple, yet distinct, HDACs [91,98]. More conclusive approaches would be to target specifically the different HDACs, while additionally considering their differential pattern of activation. In this view, class IIa HDACs are particularly striking, as their activity is under the control of synaptic activity. In pyramidal neurons, class IIa HDACs—HDAC4, 5, 7 and 9—are exported from the nucleus, and thus inactivated, upon stimulation [45,46]. Recently, it was described that in mouse spinal cord neurons, in the CFA model of chronic inflammatory pain, nociception specifically triggers only the export of HDAC4 from the nucleus of dorsal horn neurons [99]. When the nuclear export of HDAC4 was prevented, mechanical hypersensitivity was also dampened while acute pain was unaffected [99]. Next-generation RNA-sequencing of HDAC4-regulated gene expression in the dorsal spinal cord in chronic inflammatory pain brought to the identification of a novel mediator of central sensitization, the Organic Anion Transporter 1 (OAT1, encoded by the *Slc22a6* gene) [99]. Interestingly, conditional deletion of HDAC4 from primary sensory neurons primarily regulates thermal hypersensitivity [100]. Thus, it appears that HDAC4 plays a key role in chronic pain possibly with dual function in terms of regulation of sensitivity depending on the region [99,100]. Future comparative studies will hopefully clarify this aspect of HDAC4’s involvement in chronic pain which will likely be explained by specificity at the level of which genes are repressed or induced, in which cell type and under which conditions. Fewer evidence is available on the link between histone acetylation readers and chronic pain, although this might change in the nearby future as bromodomain-containing proteins have been recently linked to pain in research models [101,102].

### 3.3. Chronic Pain and ncRNAs

Of the many existing ncRNAs classes, miRNAs are the most studied group in pain research, with a constant growing number of research articles reporting on their regulation and targets in different pain states. Evidence is available for different neuropathic pain models, but also for inflammatory conditions or cancer pain. miRNAs have been associated to maladaptive changes in chronic pain at multiple levels: from the primary afferents, to the DRG, spinal cord and up to cortical regions. Further, the literature offers several examples of both upregulation and downregulation of miRNAs. In general, there seem to be a high level of specificity in which miRNAs might be regulated across different pain models, complicating the development of a generalized work model explaining the impact and functional role of miRNA in chronic pain. Specific miRNA might be upregulated in neuropathic pain but not affected or even following an opposite regulation of expression in inflammation or cancer pain. Nevertheless, few examples of common patterns of regulation across pain models exist [103]. Rather than enumerating the vast lists of which miRNAs are expressed, fueled by numerous microarray and deep-sequencing analyses, under which conditions and in which anatomical compartment [104,105], here, we will provide a brief overview of the mechanism by which miRNAs seem to influence chronic pain. Sensitization of the pain pathways is brought forward by changes in the landscape of receptors and channels ultimately resulting in hyperexcitability. Several identified or predicted target genes of miRNAs belong to this category. Indeed, miRNA targeting voltage-gated calcium channel subunits [106], voltage-gated potassium channels and their regulatory subunits [107,108], or voltage-gated sodium channels [109,110] have been found to be altered in neuropathic pain models in rats. Targets of miRNA implicated in chronic pain can also be key signaling elements or pain mediators such as NGF [111], BDNF (Brain Derived Neurotrophic Factor) [112,113,114], NF-L (Neurofilament Light Chain) [115], AKT3 (AKT Serine/Threonine Kinase 3) [116], but miRNAs might additionally influence other epigenetic regulators (i.e., MeCP2 [114], Dnmt3a [117]).

The last decade saw the rapidly growing research field of lncRNA in chronic pain. From the first identification of the voltage-gated potassium channel (Kv) Kcna2-AS-RNA, an lncRNA defined as an endogenous antisense transcript targeting the Kcna2 mRNA in neuropathic pain [118], the list of identified lncRNA associated with pain chronicity in different pain models and in humans has fast expanded [104,119,120,121]. LncRNAs have a different mode of action, as they might interact and interfere with miRNA and their processing or directly target specific molecules involved in pain processing (for detailed lists of miRNA and lncRNAs and their involvement in pain modulation, please see [104,105,119,120,121].

## 4. Conclusions and Open Questions

Different epigenetic processes are involved in all stages of pain processing and play an essential role in chronic pain. Recent years saw the exponential growth of the number of articles linking epigenetic regulators to gene expression in chronic pain. Many important mechanisms have been clarified but a comprehensive detailed view at the molecular and cellular level is far from being complete.

Although important work has been done in defining the implication of a particular epigenetic regulator on promoters of genes already known to be prominent pain modulators, what is still lacking is a systematic series of studies aimed at the untargeted identification of genes under the control of specific, distinct, epigenetic regulators in the different forms of chronic pain. As chronic pain remains a poorly managed condition at the clinical level, the importance of identification of novel targets, enabled by unbiased transcriptional profiling, carries the promise of identification of new putative therapeutic targets. What might not have been possible in the past years and necessary to tackle this aspect, namely the manipulation of expression or activity of epigenetic molecules in a time-, spatial and cell-dependent manner, is nowadays, due both to the refinement and development of new molecular biology technologies, well within reach. The combination of viral-mediated approaches, mouse genetics, and inducible promoters, as well as opto- and chemo-genetics technologies, offers tremendous possibility to specifically activate certain circuits, manipulating expression in defined cellular populations and anatomical compartments in a time-defined modality. Another important point to consider for future comparative analyses is that profiling of miRNAs or lncRNAs should be matched not only in terms of the animal and pain model used, but also time point of analyses, bioinformatic method employed and, ideally, provide information on the cell types analyzed.

A challenge in the understanding at the molecular level of what is happening along the pain pathways is that the tissues involved are highly heterogeneous, and, in addition, infiltration of additional cell types might also take place. Thus, since gene expression is a cell-type-specific characteristic, it will be important that future studies rely more on single-cell technologies and elucidate further in which cells epigenetic modulation is taking place. At present, important advances have been made in this regard, and studies relying on single-cell RNA sequencing to analyze gene expression in DRG or spinal cord in animal models of pain are gaining momentum [122,123,124,125,126,127]. Further, studies of human DRGs at the single cell level are starting to become available [128,129]. Future efforts will likely implement epigenetic mechanisms in this context and help to depict a comprehensive scenario of cell-specific epigenetic-mediated regulation of transcription in chronic pain.

Moreover, as our understanding of the areas and circuits involved in chronic pain in the brain is growing [130], future efforts should also be directed towards filling the existing gap in knowledge in terms of epigenetic modulation in such areas. In comparison to what has been described for DRGs and the spinal cord, the available information on epigenetic processes in the brain in different forms of chronic pain is minor. The difference in the amount of generated data is even more striking when considering separately the sensory and emotional aspects of pain. For the sensory component, evidence on epigenetic contributions is more than abundant and represents the vast majority of what was discussed in the present review. On the other hand, although epigenetic-centered research is also advancing in regard to the conscious dimensions of pain [131,132,133,134], it still represents quite uncharted territory, with great opportunities for important contributions to the understanding of pain.

In an attempt to establish causality between the observed epigenetic changes and hypersensitivity, an increasing number of articles have made efforts to depart from the older studies reporting mere descriptions of alterations in epigenetic marks of a particular gene or the level of ncRNA and designed elegant studies with refined strategies. These admirable efforts will hopefully be increasingly substantiated in future articles to distinguish correlative phenomena from those actually relevant to the establishment or maintenance of chronic pain.

In conclusion, while the implication of epigenetic mechanisms in chronic pain is now well accepted and some molecular and cellular mechanisms have been elucidated, the field still offers many opportunities for further in-depth investigations which will hopefully foster the development of new, more refined, therapeutic strategies for the handling of chronic pain.

## Data Availability

Not applicable.
